# Development of Thiazolidinones as Fungal Carbonic Anhydrase Inhibitors

**DOI:** 10.3390/ijms21082960

**Published:** 2020-04-22

**Authors:** Özlen Güzel-Akdemir, Simone Carradori, Rossella Grande, Kübra Demir-Yazıcı, Andrea Angeli, Claudiu T. Supuran, Atilla Akdemir

**Affiliations:** 1 Istanbul University, Department of Pharmaceutical Chemistry, Faculty of Pharmacy, 34116 Istanbul, Turkey; oguzel@istanbul.edu.tr (Ö.G.-A.); kubra.demir@istanbul.edu.tr (K.D.-Y.); 2 Dipartimento di Farmacia, “G. d′Annunzio” University of Chieti-Pescara, Via dei vestini 31, 66100 Chieti, Italy; simone.carradori@unich.it (S.C.); rossella.grande@unich.it (R.G.); 3 Neurofarba Department, Section of Pharmaceutical and Nutraceutical Sciences, Università degli Studi di Firenze, Via U. Schiff 6, 50019 Sesto Fiorentino (Florence), Italy; andrea.angeli@unifi.it (A.A.); claudiu.supuran@unifi.it (C.T.S.); 4 Centre of Advanced Research in Bionanoconjugates and Biopolymers Department, “Petru Poni” Institute of Macromolecular Chemistry, 700487 Iasi, Romania; 5 Bezmialem Vakif University, Computer-aided drug discovery laboratory, Department of Pharmacology, Faculty of Pharmacy, 34093 Istanbul, Turkey

**Keywords:** 4-thiazolidinones, antifungal activity, molecular modelling studies, carbonic anhydrases, lanosterol 14α-demethylase, CYP51a1, CgNce103

## Abstract

In our efforts to find new and selective thiazolidinone-based anti-*Candida* agents, we synthesized and tested 26 thiazolidinones against several *Candida* spp. and Gram-positive and Gram-negative bacteria. The compounds showed selective antifungal activity with potency similar to fluconazole and clotrimazole, while lacking strong antibacterial activity. Molecular docking and molecular dynamics studies were performed on *Candida* CYP51a1 and carbonic anhydrase (CA) enzymes to further suggest putative targets that could mediate the antifungal effects of these compounds. Finally, the compounds were tested in enzyme inhibition assays to assess their putative mechanism of action and showed promising *K*_I_ values in the 0.1–10 µM range against the *Candida glabrata* β-CA enzyme CgNce103.

## 1. Introduction

*Candida* species are harmless commensals in humans but can represent the most common causative agents of hospital-acquired invasive fungal infections. Indeed, *Candida* spp. are involved in opportunistic infections that cause, in immunocompromised individuals, morbidity and, in some cases, mortality, as well as an increase of the healthcare costs because of a prolonged hospitalization [1,2]. Furthermore, the incidence of the infections associated with *Candida* spp. increased over the past three decades due to the large use of immunomodulatory agents, extensive use of broad-spectrum antimicrobial drugs, and central vascular catheters [2]. *Candida albicans* represents the most common cause of invasive candidiasis in the United States of America, followed by *C. glabrata*, *C. parapsilosis*, and *C. tropicalis* [3,4]. Antifungal resistance increased over time also because of the empiric, routine prophylactic, and widespread use of antifungal-based pesticides [5].

Azole antifungal agents have been employed clinically for over 50 years; therefore, due to the development of resistant strains, the rates of mortality associated with the fungal invasive infections reached more than 50% [6,7]. The azoles are an important class of antifungal drugs that target lanosterol 14α-demethylase (CYP51a1) [8]. This enzyme plays a pivotal role in the biosynthesis pathway of ergosterol, which is a major constituent of the fungal cell membrane. However, drug resistance has become a crucial issue for this class of compounds due to their massive use as first-line therapy. *Candida* spp. developed three main strategies to become resistant to azoles: the first one is the production of multidrug pumps, which, once introduced in the fungal cell wall, allow pumping out the antimicrobial; the second one is associated with the mutation or upregulation of the genes encoding for the target enzyme promoting the alteration of the binding site, thus lowering the affinity of the drug; the third one consists in the development of an alternative pathway that is not blocked by the action of the azoles [9].

An innovative and emerging target enzyme for promising antifungal drugs and surface disinfectants are carbonic anhydrases belonging to the β-family (β-CA) and deriving from *Candida* species, such as *Candida albicans* CA (CaNce103) and *Candida glabrata* CA (CgNce103) and their ortholog ScNce103 from *Saccharomyces cerevisiae* [10,11,12]. Indeed, pathogenic yeasts can survive and proliferate in environments characterized by diverse carbon dioxide (CO_2_) concentrations accelerating the spontaneous and reversible conversion of CO_2_ to bicarbonate, subsequently used in cellular metabolism, with the help of this metalloenzyme. *C. albicans* and *Saccharomyces cerevisiae* cannot survive in atmospheric air (0.0391% of CO_2_) and in human blood (up to 5.5% of CO_2_) with a nonfunctional or inactivated carbonic anhydrase (CA, EC 4.2.1.1). Conversely, the expression of this enzyme in *C. glabrata* is regulated by the environmental CO_2_ both at the protein and transcription level but independent from the cAMP-PKA pathway [13]. Moreover, the crystal structure of recombinant CaNce103p, prepared in *Escherichia coli*, was determined at a 2.2 Å resolution [14]. It forms a zinc-containing homotetramer organized as a dimer of dimers, characterized by a slightly longer and narrower channel-like active site cavity at the bottom of which is situated the tetrahedrally coordinated zinc ion crucial for catalysis. The structural knowledge of this CA has prompted the design and synthesis of selective inhibitors, which should display a limited affinity toward mammalian α-CAs. These natural and synthetic CA inhibitors (CAIs) were active in the micromolar or nanomolar range and incorporated different chemical scaffolds such as sulphonamides [15,16,17,18,19,20,21], anions [22], carboxylic acids [23,24,25], dithiocarbamates [26], boronic acids [27,28,29], phenols [30], and phosphonamidates [31]. Recently, compounds not characterized by the presence of zinc binding groups were also shown to selectively inhibit the fungal CA isoforms in the micromolar range [32,33,34,35], leading to an opportunity to avoid cross-inhibition with structurally related human α-CAs. In the search of new chemical scaffolds, some of us proposed new thiazolidinone compounds, well-known as antimicrobial agents [36], as inhibitors of the human CAs [37], keeping in mind that this chemotype was shown to be endowed with a promising anti-*Candida* activity [38,39].

**Figure 1:**
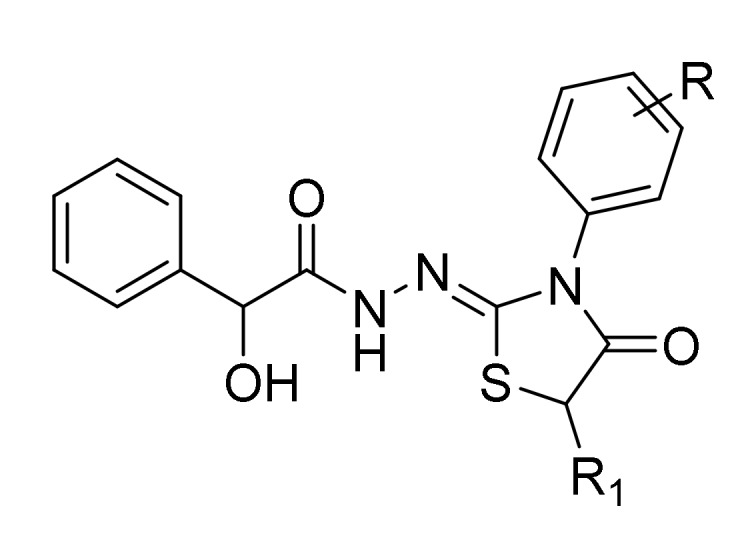
The general chemical structures of novel compounds 3a–3o and 4a–4k.

In the present work, we assessed the in vitro selective antifungal activity of previously synthesized *N*′-[3-(4-substituted phenyl)-4-oxo-1,3-thiazolidin-2-ylidene]-2-hydroxy-2-phenyl- acetohydrazide and *N*′-[3-(4-substitutedphenyl)-5-methyl-4-oxo-1,3-thiazolidin-2-ylidene] -2-hydroxy-2-phenylaceto- hydrazide derivatives (Figure 1) against sixteen clinical strains of representative *Candida* species (*C. albicans*, *C. tropicalis*, *C. parapsilosis*, *C. sakè*, and *C. glabrata*) and their putative mechanism of action based on the in vitro inhibition of the fungal β-carbonic anhydrase [37]. To further improve the knowledge of the biological potential of this scaffold, we also aimed at evaluating in vitro the additional inhibitory activity against Gram-positive and Gram-negative bacteria and in silico the peculiar binding mode and relevant interactions with fungal CYP51a1 (lanosterol α-demethylase) from *C. glabrata* and *C. albicans* and *Candida glabrata* CA (CgNce103) enzyme.

## 2. Results and Discussion

### 2.1. Microbiology

Pursuing our efforts in the discovery of innovative antifungal agents, we evaluated the susceptibility of several clinical *Candida* and bacterial species to our novel compounds by determining their minimum inhibitory concentration (MIC) by the broth microdilution method in vitro. Firstly, derivatives, dissolved in dimethylsulfoxide (DMSO), were evaluated for their antibacterial activity. Twenty-four routine clinical Gram-positive (*Staphylococcus aureus* and *Staphylococcus epidermidis*) and Gram-negative isolates (*Escherichia coli* and *Enterobacter* spp.) were tested following the experimental procedures previously reported [38]. Then, the antifungal activity was evaluated against sixteen clinical fungal isolates of the most relevant *Candida* spp. (*C. albicans*, *C. tropicalis*, *C. parapsilosis*, *C. sakè*, and *C. glabrata*) and compared with topical and systemic reference drugs clotrimazole and fluconazole, respectively (Table 1).

All compounds did not display any antibacterial activity against Gram-positive or Gram-negative clinical isolates (MIC ≥ 256 μg/mL), disregarding the substitution pattern and with respect to the standard drug ceftazidime (MIC = 4–8 μg/mL). A slightly better inhibitory activity versus *S. epidermidis* strains can be observed for compounds 4c, 4d, 4i, and 4j characterized by the presence of R_1_ = CH_3_ on the C5 of the thiazolidinone nucleus (MIC = 128 μg/mL). Thus, the further comparison with the results for antifungal activity in Table 1 suggested a selective action restricted to *Candida* spp., as corroborated by the inefficacy against bacterial species. Comparing the two series of derivatives (3 and 4), the introduction of a methyl moiety at the C5 of thiazolidinone usually led to lower MIC values against the five fungal species, whereas the C5-unsubstituted thiazolidinones were poor inhibitors of the growth of *C. tropicalis*, *C. parapsilosis*, and *C. sakè*. Among the compounds belonging to series 3 (R_1_ = H), derivatives 3a-3c (R = 2-F, 3-F, and 4-F, respectively) and 3f (R = 4-Cl) displayed the most interesting inhibitory activity (MIC = 4–8 μg/mL) against all *C. albicans* strains. Conversely, other substituents such as Br, CH_3_, OCH_3_, NO_2_, and CF_3_ furnished less-active compounds (MIC range values of 8–32 μg/mL). Against *C. glabrata* strains, compounds 3f (R = 4-Cl), 3i (R = 4-Br), and 3o (R = 3-CF_3_) were the best-in-class inhibitors, showing the lowest MIC range values (4–8 μg/mL) within this series. Compound 3o was also effective in the same MIC range against *C. sakè* strains. On the contrary, series 3 was almost weak versus both *C. tropicalis* and *C. parapsilosis* strains.

The antifungal results for series 4 (R_1_ = CH_3_) furnished a more promising scenario. Collectively, they displayed a better inhibitory profile toward all the *Candida* species under investigation, with MIC range values very similar to the reference drugs clotrimazole and fluconazole (2 μg/mL). The presence of a halogen (F, Cl, and Br) on the aryl ring at N3 of the thiazolidinone nucleus induced a remarkable growth inhibition against all *Candida* spp. and, in particular, versus *C. sakè*, *C. glabrata*, and *C. tropicalis* (MIC = 4–8 μg/mL). The most interesting results were registered for compounds having a Cl, Br, or CH_3_ in the *meta* or *para* position of the aryl ring (4d–4g). Conversely, compounds with other substitution patterns were shown to be less potent.

Collectively, this cell-based evaluation suggested that thiazolidinone compounds can be regarded as promising selective antifungal agents toward different *Candida* species. The presence of several chemical groups on the core nucleus could further suggest a proper modulation of the pharmacokinetic and chemical-physical properties suitable for the selection of lead compounds to be clinically investigated.

### 2.2. Molecular Modeling Studies to Suggest Putative Targets

To suggest possible targets that mediate the antifungal activity of the thiazolidinones, we performed docking studies against *Candida* CYP51a1 and CA enzymes, followed by molecular dynamics simulations.

#### 2.2.1. Molecular Modeling Studies of *C. albicans* and *C. glabrata* CYP51a1 Enzymes

Crystal structures of *Candida albicans* (CaCYP51a1; PDB: 5v5z; 2.9 Å) and *Candida glabrata* (CgCYP51; PDB: 5jlc; 2.4 Å) CYP51 enzymes both in complex with itraconazole have been obtained from the RCSB Protein Data Bank. The sequence identity between both enzymes is approximately 64%. However, the sequence identity in the active site (defined as all amino acids within 4.5 Å of itraconazole) is 90%. The overall identity similarity of CaCYP51a1 to the other *Candida* CYP51 enzymes is between 64–84%, while the sequence identity in the active site is 90–95%.

**Figure 2:**
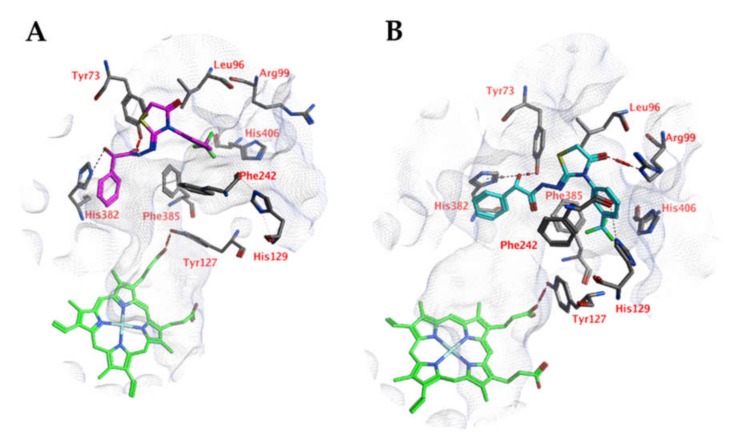
(**A**) The docked pose of compound 4o (turquoise) in the active site of CgCYP51 (PDB: 5jlc). (**B**) The docked pose of compound 4o (purple) in the active site of CgCYP51 after a 5-ns molecular dynamics simulation. The haem group is indicated in green sticks. The pocket surface is indicated with a white mesh. Hydrogen bonds are indicated in red dashed lines. H-arene interactions are indicated in yellow dashed lines.

Docking studies into the active site of CgCYP51a1 did not reveal any poses in which the ligand could directly interact with the iron atom of the haem group. Instead, poses have been obtained in which the ligands are located near the entrance of the cavity (Figure 2A). For example, compound 4o forms a hydrogen bond with the side chain of Arg99 via its carbonyl group. The hydroxyl group (*S* isomer) of the ligand is able to form hydrogen-arene interactions with His382 and a hydrogen bond with Tyr73. This interaction is not possible for the *R* isomer of the compound. Cation-π interactions are possible between the substituted phenyl group of the ligand and the side chain of Arg99. Hydrophobic interactions are formed with Tyr73, Leu96, Leu97, Phe242, Val243, His382, and Phe385. Other compounds of these series can adopt similar docked poses.

**Figure 3:**
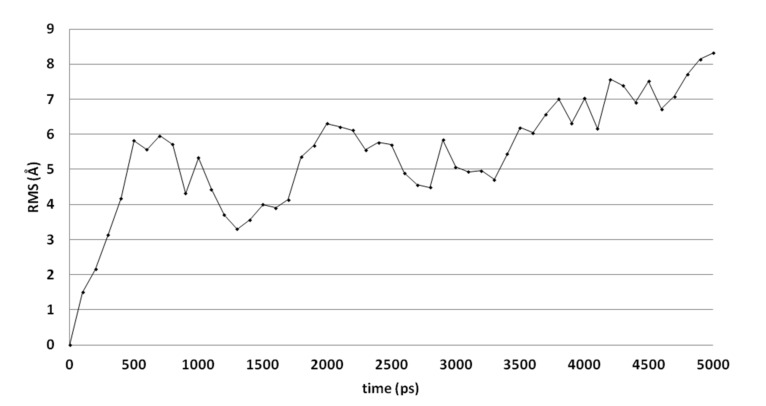
The RMSD of the ligand heavy atoms during the 5-ns molecular dynamics simulation of compound 4o in the active site of CgCYP51.

Molecular dynamics (MD) simulations indicate that this docked pose is not stable as the ligand RMSD value increases to levels above 3 Å (Figure 3). Early during the simulation (200 ps), the hydrogen bond between the ligand and Arg99 is lost due to the adoption of another sidechain conformation. As a result, the ligand moves closer to His382 to result in a new binding pose (already observed at 300 ps). The ligand hydroxyl group forms a hydrogen bond with His382 (86% of the 5 ns simulation), while the ligands carbonyl group forms a hydrogen bond with Tyr73 (Figure 2B). This hydrogen bond is observed during 94% of the 5 ns unconstrained simulation. The peptide bond and the double bond of the ligand are not in plane (bond between the two adjacent nitrogen atoms), while this was expected for the sp^2^ hybridized system. A DFT calculation (ωB97X-D functional and 6-311+G** basis set) was performed using Spartan ′18 (v1.4.0, WaveFunction Inc., CA, USA) to investigate the bond order between the two adjacent nitrogen atoms, and this was found to be 0.89 (Mulliken bond order). This indicates that the obtained ligand conformation could be possible. Many compounds of series 3 and 4 show similar docked poses, as described for compound 4o (Figure 2A).

**Figure 4:**
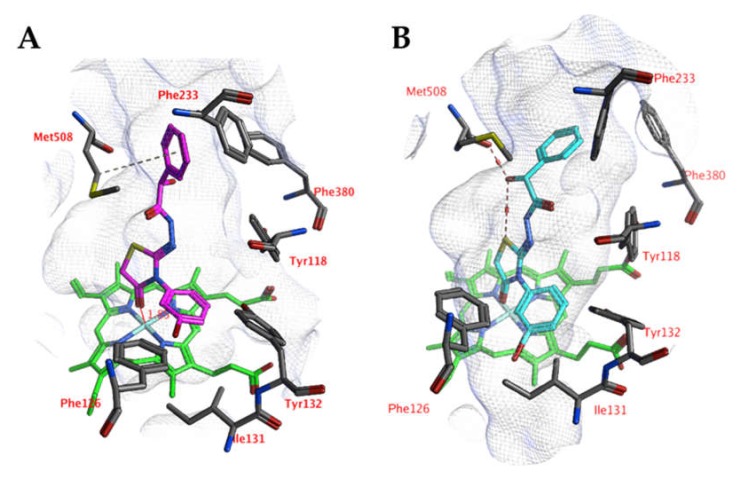
(**A**) The docked pose of compound 4h (turquoise) in the active site of CaCYP51 (PDB: 5v5z). (**B**) The docked pose of compound 4h (purple) after a 5-ns molecular dynamics simulation. The haem group is indicated in green sticks. The pocket surface is indicated with a white mesh. Hydrogen bonds are indicated in red dashed lines.

**Figure 5:**
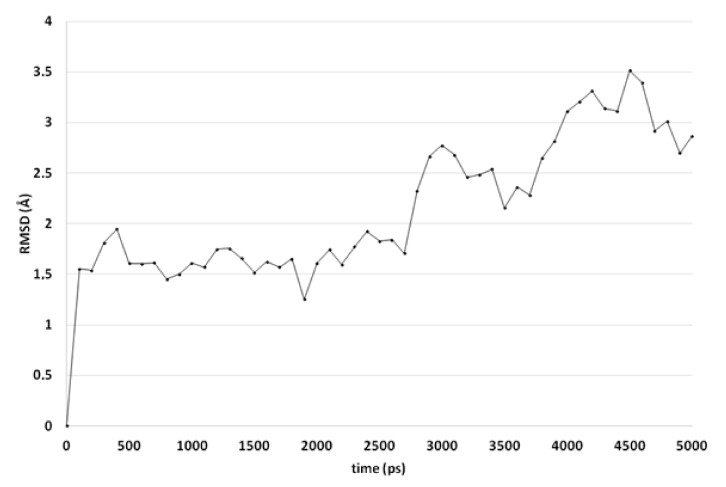
The RMSD of the ligand heavy atoms during the 5-ns molecular dynamics simulation of compound 4h in the active site of CaCYP51.

A comparable docked pose, as observed for compound 4o in the active site of CgCYP51a1 (Figure 2A), has not been observed in the active site of CaCYP51a1 due to the change of Arg99 (CgCYP51a1) to Lys90 (CaCYP51a1). Instead, the ligands enter deeper into the active site, and several ligands form interactions with the haem iron via their carbonyl group (Figure 4, compound 4h). The substituted phenyl group forms hydrophobic interactions with the haem group. The hydroxyl group (*R* isomer) forms a hydrogen bond with the carbonyl backbone of Met508 and an intramolecular interaction with the ligand sulfur atom. MD simulations indicate that the interaction between the ligand’s carbonyl group and the haem iron is stable during the 5-ns simulation. The intramolecular bond between the hydroxyl group and the sulfur atom is lost early during the simulation (100 ps), while the hydrogen bond with the backbone carbonyl of Met508 is stable only during the first 2700 ps of the simulation. This can also be observed as the RMSD of the ligand heavy atoms increases after 2700 ps (Figure 5). During the MD simulation, a hydrogen bond between the ligand and the backbone carbonyl of Met508 is observed during 70.6% of the 5-ns simulation, while a hydrogen-arene interaction between the sidechain of Met508 and the unsubstituted phenyl group is observed during 9.8% of the simulation. Similar docked poses have been observed for the other compounds of series 3 and 4.

**Figure 6:**
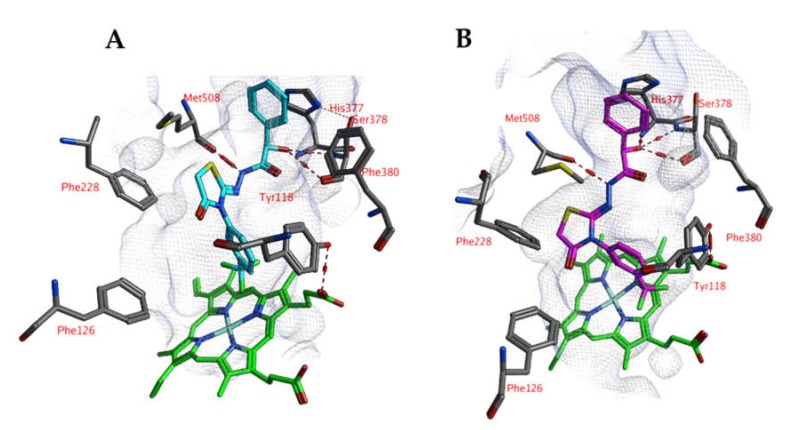
(**A**) The docked pose of compound 4o (purple) in the active site of CaCYP51 (PDB: 5v5z). (**B**) The docked pose of compound 4o (turquoise) after a 5-ns molecular dynamics simulation. The haem group is indicated in green sticks. The pocket surface is indicated with a white mesh. Hydrogen bonds are indicated in red dashed lines.

**Figure 7:**
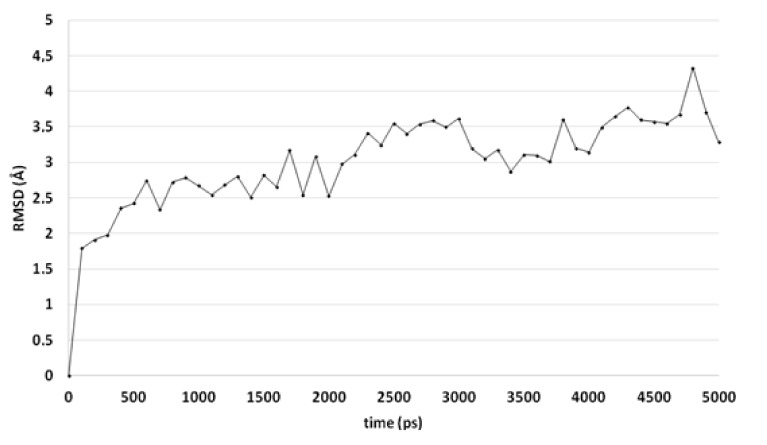
The RMSD of the ligand heavy atoms during the 5-ns molecular dynamics simulation of compound 4o in the active site of CaCYP51.

An alternative docked pose in CaCYP51a1 has been observed for compound 4o (Figure 6). Hydrogen bonds are observed between the ligand and the backbones of Ser378 and Met508. The trifluorophenyl group is located close to the haem group and forms hydrophobic interactions. MD simulations result in RMSD values around 2.5–3.5 Å for most of the simulation and indicate that the docked pose is stable (Figure 7). Only the substituted phenyl group is dynamic, as it only forms hydrophobic interactions with haem. The hydrogen bonds with Ser378 and Met508 are not lost during the simulation. The hydrogen bond with the backbone of Met508 is present during the whole duration of the simulation, while the hydrogen bond to the backbone of Ser378 is present during 9.2%.

The residues in the active site that are in direct contact with ligands 4h and 4o (CaCYP51a1) are conserved in both enzymes, but they have a slightly different conformation. Therefore, these poses have not been observed in CgCYP51a1, but they may be still possible after induced fit effects.

#### 2.2.2. Molecular Modeling Studies of the *Candida glabrata* β-CA Enzyme

**Figure 8:**
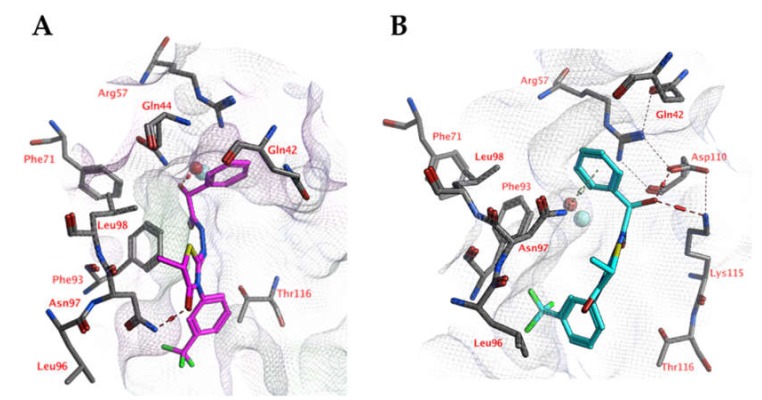
(**A**) The docked pose of compound 5k (purple) in the active site of CgNce103 (homology model). (**B**) The docked pose of compound 5k (turquoise) after a 5-ns molecular dynamics simulation. The pocket surface is indicated with a white mesh. Hydrogen bonds are indicated in red dashed lines. The zinc ion is indicated in a turquoise sphere. The water molecule is indicated in a red sphere.

Docking studies indicate that the ligands may interact with the zinc-bound water molecule, as shown for compound 5k (Figure 8). This interaction is only possible if the ligand’s hydroxyl group is in the *R* isomeric conformation. The unsubstituted phenyl group forms a cation-π interaction with Arg57. The ligand’s peptide bond is near-linear and water-exposed and does not interact with the protein. The carbonyl group of the ligand forms an additional hydrogen bond with the side chain of Asn97. Hydrophobic interactions are formed with Phe93.

**Figure 9:**
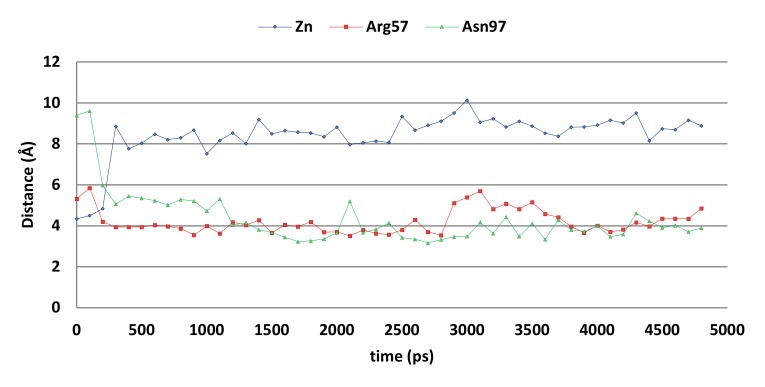
The distances between compound 5k and the zinc ion (blue), Arg57 sidechain (red), and Asn97 side chain (grey) of the CgNce103 active site during the 5-ns MD simulation.

However, molecular dynamics simulations indicate that the docked pose may not be stable (Figure 9). The overall distance between the ligand’s hydroxyl group and the active site zinc ion increases significantly during the first 500 ps of the simulation, while the distances between the ligand’s unsubstituted phenyl group (centroid) to the Arg57 side chain (atom Cz) and Asn97 side chain (atom ND2) decreases. As a result, cation-π interactions with Arg57 and hydrogen-arene interactions with Asn97 can be formed after 500 ps.

Molecular modeling studies shed light on the putative targets of these compounds, highlighting how the introduction of specific chemical moieties in the thiazolidinone scaffold can efficiently contribute to a stronger interaction with well-established and crucial targets for the growth of the fungal cell. Moreover, the impact of the stereochemistry on the enzyme-inhibitor adducts pointed out that both the enzymes can stereoselectively recognize the substrates (S isomer for CaCYP51a1 and R isomers for CgCYP51a1 and CgNce103).

### 2.3. Enzyme Inhibition Studies of CgNce103 Compared to hCA I and II

To better understand the putative mechanism of action of this scaffold and to confirm the in silico data, the thiazolidinones were also screened by means of a stopped-flow CO_2_ hydrase assay with the aim of assessing their potential to inhibit *C. glabrata* carbonic anhydrase (CgNce103) (Table 2). This target has been recently recognized as important for the fungal growth in CO_2_-enriched environments. Among the chemotypes designed for its inhibition in the literature, it was proposed and widely validated the use of zinc binding groups (e.g. sulfonamides and their bioisosteres, carboxylates and their derivatives, phenols, and so on). Thiazolidinones were recently assayed as potent antifungal agents [39,40], and some of them were tested as carbonic anhydrase inhibitors in order to discover and develop innovative mechanisms of inhibition [37,41]. From the data reported in Table 2, it is possible to extrapolate a very remarkable selectivity profile of our thiazolidinones against this fungal β-CA over the two ubiquitous human isoforms (hCA I and II) being the selectivity index (SI) for compound 4h ˃1000 (expressed as the ratio between *K*_I_ CA I or II and *K*_I_ CgNce103 values) and ˃100 for most of the other compounds. This aspect is very useful for medicinal chemists to design the structural requirements, providing selectivity among the large family of CA isozymes. Collectively, all the compounds (series 3 and 4) were inactive against hCA I (*K*_I_ ˃100 μM) and poorly active against hCA II (*K*_I_ ˃ 47.4 μM). On the contrary, the inhibition profile versus CgNce103, except for two derivatives (3g, *K*_I_ CgNce103 = 10.2 μM and 3l, *K*_I_ CgNce103 = 6.30 μM), was characterized by nanomolar *K*_I_ values. In more detail, compounds with R_1_ = H were slightly less efficient with respect to their counterparts with R_1_ = CH_3_. Compound 3o (*K*_I_ = 0.12 μM) was the best representative of this series. Among 4a–4k, there were several examples of potent nanomolar inhibitors, usually characterized by an electron-withdrawing *meta*- or *para*-substituent on the aryl ring at N3 of the core nucleus (4a, 4g, 4h, and 4k). Moreover, these compounds, despite being ten times less potent than acetazolamide (AAZ, *K*_I_ = 0.011 μM) against CgNce103, displayed a better selectivity index toward human α-CAs with respect to the same reference drug.

This information confirmed that sulfonamide-containing compounds are usually good CA inhibitors but endowed with limited isoform selectivity. Thus, our new scaffold can be further exploited for the design of new antifungal agents with the potential to inhibit selectively the fungal isoforms over human ones. Indeed, keeping in mind the structure-activity relationships extrapolated for this scaffold, researchers can exploit the structural requirements to display such biological activity. Moreover, our best-in-class compounds have an important significance for further clinical development, because their CA-based mechanism of action and isoform selectivity over human CAs could propose them as lead compounds for in vivo studies of fungal infections. These compounds can be used as coadjuvant or alternative therapy to classical azoles, limiting their dose, emerging resistance, and side effects.

## 3. Materials and Methods

### 3.1. Bacterial and Fungal Strains and Culture Conditions

The antimicrobial activity of these thiazolidinones was evaluated versus 24 clinical Gram-positive (*S. aureus* and *S. epidermidis*) and Gram-negative (*E. coli* and *Enterobacter* spp.) microorganisms. The antifungal activity of these derivatives was then tested versus 16 clinical fungal isolates belonging to the most relevant *Candida* spp. (*C. albicans*, *C. tropicalis*, *C. parapsilosis*, *C. sakè*, and *C. glabrata*). Prior to testing, each isolate was cultured on a selective medium to ensure purity and optimal growth, as previously reported [38]. The isolates were routinely identified by the morphological aspects of colonies and by biochemical identification. The used clinical isolates were collected from specimens of patients at the ‘Azienda Policlinico Umberto I’ (Sapienza University of Rome) and were obtained from hematology/oncology and surgery departments, which also included an intensive care unit. In particular, the samples were isolated from the upper and lower respiratory tract, blood, and indwelling venous catheters; the isolates were identified by conventional methodologies. Participating subjects gave their written informed consent for this study.

### 3.2. Antibacterial Activity

The minimum inhibitory concentration (MIC) was determined by using the broth microdilution method in 96-well polystyrene microtitre plates (Eppendorf, Hamburg, Germany) according to the Clinical & Laboratory Standards Institute (CLSI) [42] guidelines. Briefly, bacterial overnight broth cultures were resuspended in Mueller–Hinton broth (Microbiology Systems, Cockeysville, MD, USA) to an optical density at 550 nm (OD_550_) of 0.8 corresponding to 1–5 × 10^8^ colony-forming units (CFU)/mL. The broth cultures were subsequently diluted in Mueller–Hinton Broth to yield the appropriate density corresponding to 1–5 × 10^5^ CFU/mL per well in a 100-μL final volume. All derivatives were dissolved in DMSO and filtered through 0.22-µm cellulose membrane filters (Corning, New York, NY, USA). Serial dilutions of each compound ranging from 128 to 0.5 μg/mL were added to each well. The plates included controls consisting of (i) bacteria without the addition of the compound, (ii) just the medium, and (iii) just the compound. Ceftazidime was used as a reference drug for comparison. The plates were incubated for 18 to 22 h at 37 °C. The minimum inhibitory concentration (MIC) for all isolates was defined as the lowest concentration of antibacterial agent that completely inhibited the growth of the organism, as detected by the unaided eye. Three independent experiments were performed in triplicate.

### 3.3. Antifungal Activity

The in vitro antifungal activities were determined by the broth microdilution method with sabouraud dextrose broth (BBL Microbiology Systems, Cockeysville, MD, USA), as recommended by the by the CLSI [43]. Ninety-six-well polystyrene microtitre plates containing serial dilutions of each compound were inoculated with each microorganism to yield the appropriate density (1–5 × 10^3^ cells/mL) in a 100-μL final volume. Each plate included (i) fungi without the addition of the compound, (ii) just the medium, and (iii) just the compound. Topical and systemic antifungal reference drugs, clotrimazole and fluconazole, were inserted in the experiment. The plates were incubated for 24 h at 35 °C. The MIC for all isolates was defined as the lowest concentration of the antifungal agent that completely inhibited the growth of the organism, as detected by the unaided eye. Three independent experiments were performed in triplicate.

### 3.4. Molecular Modeling Studies

#### 3.4.1. Preparation of Ligand Structures

Three-dimensional structures of all ligands, including all their stereoisomers, were prepared, and the most prevalent protonation state of the ligands at pH 7 were calculated using the MOE Software package (v2019.0102, Chemical Computing Group, Inc, Montreal, QC, Canada). The ligands were energy minimized using a steepest-descent protocol (MMFF94x force field).

#### 3.4.2. Preparation of CaCYP51a1 and CgCYP51a1 Crystal Structures

Crystal structures of *Candida albicans* (CaCYP51a1; PDB: 5v5z; 2.9 Å) and *Candida glabrata* (CgCYP51a1; PDB: 5jlc; 2.4 Å) CYP51a1 enzymes, both in complex with itraconazole, have been obtained from the RCSB Protein Data Bank. Buffer molecules, ions, and water molecules were deleted if present (only in 5jlc), and the protein atoms, haem atoms, and iron atoms were retained. Hydrogen atoms were added according to the protonate 3D protocol found in the MOE package [44]. Subsequently, the structures were subjected to energy minimization using a steepest-descent protocol (applied (AMBER14:EHT force field).

#### 3.4.3. Preparation of CgNce103 Homology Models

Homology models of CgNce103 (GenBank: CAG59355.1; 219 amino acids) were constructed using the crystal structure of the green alga *Coccomyxa* CA in complex with acetazolamide (CmCA; 3ucj; 1.85 Å) as previously described (50 models) using the MOE Software package [34]. The homology model with the highest contact score was selected, and steepest-descent energy minimization protocols were applied (AMBER14:EHT force field). All heavy atoms of acetazolamide (acquired from the 3ucj structure), the active site residues (all residues within 4.5 Å of acetazolamide), the zinc ion, the zinc-binding His residues, and the protein backbone were fixed, and the other parts were minimized using a controlled release of position restraints. The minimized structure was used in the docking studies.

### 3.5. Docking Studies

Docking calculations were performed using the FlexX docking tool (v2.3.2; BioSolveIT GmbH, St. Augustin, Germany) within MOE. The binding pocket was defined as all residues within 6.5 Å of the reference ligand acetazolamide (CgNce103 homology model) or itraconazole (CaCYP51a1 and CgCYP51a1 crystal structures). All ligands were docked fifty times, and the best-scoring three poses were subjected to refinement calculations. To this end, the docked ligand and binding pocket residues (defined as all residues within 6.5 Å of the ligand) were energy-minimized and rescored using the GBVI/WSA force field [45].

### 3.6. Molecular Dynamics Simulations

All molecular dynamics simulations were performed using the Yasara Structure software package (v18.8.9, YASARA Biosciences GmbH, Vienna, Austria) [46,47]. The selected docked poses (ligand-enzyme complexes) were first placed into the center of a cuboid box with periodic boundary conditions (minimal distance of 10 Å between the protein and boundary). Afterwards, both water molecules (density: 0.997 gr/mL) and counter ions (NaCl) were added to generate a solvated and neutral system. The system was energy-minimized using a steepest-descent protocol (AMBER14). The system was first heated from 0 K to 300 K during a 100-ps followed by a 200-ps equilibration simulation (position restraints on all protein and ligand heavy atoms). Finally, the system was simulated for 5 ns at a constant temperature (300 K, Berendsen, default values) and pressure (1 bar, Berendsen, default values), without any position restrains (production run). The only restraints applied were distance restraints to keep the zinc ion in the correct orientation towards Cys53, His108, and Cys111 (default settings). The timestep was set to 2 × 1.25 fs, and all bonds were constrained using the LINCS algorithm. Snapshots were taken every 100 ps of the 5-ns production run.

### 3.7. Enzyme Inhibition Assays

An applied photophysics stopped-flow instrument has been used for assaying the fungal and human CA-catalyzed CO_2_ hydration activity [48]. Phenol red (0.2 mM) has been used as the indicator, working at the absorbance maximum of 557 nm, with 20-mM HEPES (pH 7.5 for α-CAs) or TRIS (pH 8.3 for the β-CA) as buffers and 20-mM NaClO_4_ (for maintaining constant the ionic strength), following the initial rates of the CA-catalyzed CO_2_ hydration reaction for a period of 10–100 s. The CO_2_ concentrations ranged from 1.7 to 17 mM for the determination of the kinetic parameters and inhibition constants. In particular, CO_2_ was bubbled in distilled deionized water for 30 min until saturation. A CO_2_ kit (Sigma-Aldrich, Milan, Italy) was used to measure the concentration in serially diluted solutions from the saturated one at the same temperature. For each inhibitor, at least six traces of the initial 5–10% of the reaction have been used for determining the initial velocity. The uncatalyzed rates were determined in the same manner and subtracted from the total observed rates. Stock solutions of the inhibitor (1 µM) were prepared in distilled-deionized water, and dilutions up to 0.1 nM were done thereafter with the assay buffer. Inhibitor and enzyme solutions were preincubated together for 15 min at room temperature prior to the assay in order to allow for the formation of the E-I complex or for the eventual active site-mediated hydrolysis of the inhibitor. The inhibition constants were obtained by the nonlinear least-squares methods using PRISM 3 and the Cheng-Prusoff equation and represent the average from at least three different determinations. All recombinant CA isoforms were obtained in-house, as previously reported [49,50].

## 4. Conclusions

We synthesized and tested 26 new thiazolidinones against *Candida* spp. and several Gram-positive and Gram-negative bacteria. The compounds showed selective antifungal activity, while lacking strong antibacterial activity. Molecular docking and molecular dynamics studies were performed on *Candida* CaCYP51a1, CgCYP51a1, and CgNce103 enzymes in order to evaluate the putative mechanism of action. For CYP51a1 enzymes, the thiazolidinones could bind both to the entrance of the active site, as well as in the active site. In the latter case, the compounds were able to form an interaction with the haem iron atom. For CgNce013, no docked poses were found in which the ligands directly interacted with the active-site zinc ion. Docked poses in which the ligand forms interactions with a zinc-bound water molecule were not stable during 5-ns molecular dynamics simulations. Nevertheless, the ligands may be able to bind close to the zinc-bound water molecule without a direct interaction, while they form hydrogen-arene interactions with Asn97 and cation-π interactions with Arg57. CgNce103 enzyme inhibition assays revealed that these thiazolidinones indeed inhibit this fungal CA enzyme with *K*_I_ values in the 0.1–10 µM range. Collectively, this new class of compounds can be further developed as innovative antifungal agents endowed with an alternative mechanism of action with respect to azoles.

## Figures and Tables

**Table 1:** Minimum inhibitory concentration (MIC) of newly synthesized compounds (3a–3o and 4a–4k), clotrimazole, and fluconazole against sixteen clinical strains of *Candida* species.

	R	R_1_	Tested Fungi (MIC Values μg/mL)				
			*C. albicans* 5 strains	*C. sakè* 3 strains	*C. glabrata* 3 strains	*C. tropicalis* 3 strains	*C. parapsilosis* 2 strains
3a	2-F	H	4–8	16	16	32	32
3b	3-F	H	4–8	16	16	32	32
3c	4-F	H	4–8	16	16	32	32
3d	2-Cl	H	8–32	8–32	8–32	32	32
3e	3-Cl	H	8–32	8–32	8–32	32	32
3f	4-Cl	H	4–8	16	4–8	32	16
3g	2-Br	H	8–16	16	8–32	8–32	8
3h	3-Br	H	8–16	16	8–32	8–32	8
3i	4-Br	H	8–16	16	4–8	16	16
3j	2-CH_3_	H	8–16	8–32	8–32	32	16
3k	3-CH_3_	H	8–32	8–32	8–32	32	16
3l	4-CH_3_	H	8–32	8–32	8–32	32	32
3m	4-OCH_3_	H	8–32	8–32	8–32	32	32
3n	4-NO_2_	H	8–32	8–32	8–32	32	32
3o	3-CF_3_	H	16–32	4–8	4–8	32	32
4a	3-F	CH_3_	16–32	4–8	8–32	8–32	8
4b	4-F	CH_3_	16–32	4–8	8–32	8–32	8
4c	3-Cl	CH_3_	16–32	4–8	8–32	8–32	8
4d	4-Cl	CH_3_	4–16	4–8	8–32	8–32	8
4e	3-Br	CH_3_	4–16	4–8	4–8	4–8	32
4f	4-Br	CH_3_	4–16	4–8	4–8	4–8	32
4g	3-CH_3_	CH_3_	4–16	4–8	4–8	4–8	32
4h	4-CH_3_	CH_3_	8–32	8–32	8–32	16	32
4i	4-OCH_3_	CH_3_	8–32	8–32	8–32	16	32
4j	4-NO_2_	CH_3_	8–32	8–32	8–32	16	32
4k	3-CF_3_	CH_3_	32–128	8–32	8–32	128	64
Clotrimazole			2	2	2	2	2
Fluconazole ^a^			2	2	2	2	2

^a^ All the *Candida albicans*, *tropicalis*, and *parapsilosis* strains were sensitive to fluconazole, *Candida glabrata* strains resulted to be intermediate to fluconazole, and, for *C. sakè* strains, there were no data according to the Clinical Breakpoints for Fungi v. 10.0 of the Eucast Guidelines (valid from 4 February 2020).

**Table 2:** Inhibition data of selected human and fungal CA isoforms (hCA I, hCA II, and CgNce103) by the most promising thiazolidinone compounds and the standard sulfonamide inhibitor acetazolamide (AAZ) by a stopped-flow CO_2_ hydrase assay.

	*K*_I_ * (μM)		
	hCA I	hCA II	CgNce103
3a	>100	>100	0.27
3b	>100	>100	0.48
3c	>100	76.7	0.38
3d	>100	>100	0.32
3e	>100	50.3	0.43
3f	>100	62.9	0.51
3g	>100	>100	10.2
3h	>100	55.7	0.82
3i	>100	47.4	0.87
3k	>100	79.3	0.41
3l	>100	>100	6.30
3m	>100	>100	0.39
3o	>100	82.8	0.12
4a	>100	>100	0.11
4b	>100	>100	0.48
4c	>100	>100	0.12
4e	>100	68.2	0.56
4f	>100	>100	0.11
4g	>100	>100	0.50
4h	>100	>100	0.09
4i	>100	>100	0.69
4k	>100	>100	0.11
AAZ	0.25	0.012	0.011

* Mean from 3 different assays, by a stopped-flow technique (errors were in the range of ± 5–10% of the reported values).
